# Restricted Feeding Resets the Peripheral Clocks of the Digestive System

**DOI:** 10.3390/biomedicines11051463

**Published:** 2023-05-17

**Authors:** Kazuo Nakazawa, Minako Matsuo, Naobumi Kimura, Rika Numano

**Affiliations:** 1 Department of Applied Chemistry and Life Science, Toyohashi University of Technology, Toyohashi 441-8580, Japan; 2 Institute for Research on Next-Generation Semiconductor and Sensing Science, Toyohashi University of Technology, Toyohashi 441-8580, Japan

**Keywords:** circadian rhythm, *Period 1 luciferase* transgenic mice, restricted feeding, phase shift

## Abstract

All organisms maintain an internal clock that matches the Earth’s rotation over a period of 24 h, known as the circadian rhythm. Previously, we established *Period1 luciferase* (*Per1::luc*) transgenic (Tg) mice in order to monitor the expression rhythms of the *Per1* clock gene in each tissue in real time using a bioluminescent reporter. The *Per1* gene is a known key molecular regulator of the mammalian clock system in the autonomous central clock in the suprachiasmatic nucleus (SCN), and the peripheral tissues. *Per1::luc* Tg mice were used as a biosensing system of circadian rhythms. They were maintained by being fed ad lib (FF) and subsequently subjected to 4 hour (4 h) restricted feeding (RF) during the rest period under light conditions in order to examine whether the peripheral clocks of different parts in the digestive tract could be entrained. The peak points of the bioluminescent rhythms in the *Per1::luc* Tg mouse tissue samples were analyzed via cosine fitting. The bioluminescent rhythms of the cultured peripheral tissues of the esophagus and the jejunum exhibited phase shift from 5 to 11 h during RF, whereas those of the SCN tissue remained unchanged for 7 days during RF. We examined whether RF for 4 h during the rest period in light conditions could reset the activity rhythms, the central clock in the SCN, and the peripheral clock in the different points in the gastrointestinal tract. The fasting signals during RF did not entrain the SCN, but they did entrain each peripheral clock of the digestive system, the esophagus, and the jejunum. During RF for 7 days, the peak time of the esophagus tended to return to that of the FF control, unlike that of the jejunum; hence, the esophagus was regulated more strongly under the control of the cultured SCN compared to the jejunum. Thus, the peripheral clocks of the digestive system can entrain their molecular clock rhythms via RF-induced fasting signals in each degree, independently from the SCN.

## 1. Introduction

The suprachiasmatic nucleus (SCN) of the hypothalamus maintains the mammalian circadian clock system and is the central regulator of the circadian rhythm of all peripheral tissues in an individual. The single-cell molecular clock is driven by the transcription and translation feedback loops of several clock genes, such as *Period1* (*Per1*) and *Period2* (*Per2*) [1,2,3,4]. Every morning, photo signals from the retina transiently induce the transcription of photoreactive clock genes, such as *Per1* and *Per2*, in the SCN, resetting the phase of the circadian clock [5,6]. The feeding time of mice reportedly affects the circadian clock phase of behavior and certain peripheral tissues [7,8,9].

Previously, we established *Per1::luc* transgenic (Tg) mice, wherein the *Per1* promoter region (6.8 kb) was fused with a firefly luciferase reporter for the real-time monitoring of *Per1* expression and the molecular rhythms in each tissue, as *Per1* is a key molecular regulator in the mammalian core clock system [6,7]. Using these biosensing systems, and subjecting *Per1::luc* Tg mice to 4 h restricted feeding (RF) during the daytime, which is the resting time of nocturnal mice, we can reset the phase of *Per1::luc* emission rhythms in peripheral tissues, such as those of the liver, under constant dark conditions [10]. The RF-induced nonphotic signals ensure consistent circadian rhythms, independently from the SCN central clock, which is synchronized via photic signals. Some peripheral tissues can be moderately influenced via certain nonphotic signals, such as food intake [8,9,10,11,12,13,14] and glucocorticoids [15].

Limiting the feeding time of the mice to only during the short rest period under light conditions induces food anticipatory activity (FAA), which involves increased behavioral activity before the feeding time. The circadian clock phase in the behavioral activities and peripheral tissues, such as those of the liver, were reset by limiting the feeding time; however, the circadian clock phase was not reset in the SCN [10,11,12,13,14]. The desynchronization in the central and peripheral clocks that was induced by a limited feeding time engendered lifestyle-related diseases, such as obesity and diabetes. Therefore, a normal relationship between the peripheral clocks is essential to adjust the induced clock system in all of the organs [16,17,18].

Herein, *Per1::luc* Tg mice were employed as biosensors in order to compare the ability to reset the circadian rhythm of each peripheral tissue in response to inducible environmental signals. Furthermore, by culturing each organ sample collected from identical *Per1::luc* Tg mice, the rhythm of each organ from the same individual could be simultaneously observed. Conversely, in a single *Per1::luc* Tg mouse that was used as a biosensor, the circadian rhythm could be estimated as a time course of single-consecutive rhythmic data without individual variations. Previously, we developed *Per1::luc* Tg rats by using the same DNA construct. Both the central and the peripheral clocks in the SCN and the peripheral tissues could be monitored as *Per1* rhythmic bioluminescent oscillation from the same individual. In a previous study, the phase shift ability of the SCN and the liver was compared during 4 h RF using *Per1::luc* Tg rats [10]. The circadian clock peak time in the liver was found to be entrained from the second day of using the 4 h RF under light–dark conditions, which was shifted to 10 h, alongside the FAA [10]. Conversely, the 4 h RF did not shift the peak time of the SCN, as the feeding time signal could not reset the phase of the central clock in the SCN [10]. Additionally, the phase of the *Per1::luc* bioluminescent rhythms in the stomach and the colon of the digestive system was shifted for 2 days to a diurnal peak during RF, which returned to a nocturnal peak when they were fed ad lib (FF) for ~5 days [19]. Therefore, it is possible that the liver was more strongly entrained by the feeding clock, and the stomach and the colon were more affected by the central clock. There is a complex correlation between the feeding clocks, the peripheral tissues, the photic clock, and the SCN [20,21,22,23]. The transcriptional feedback loop of clock gene, which is essential for the function of the circadian clock, was not indispensable to FAA, because mice with mutated clock genes exhibited normal FAA [24,25,26].

Herein, we examined whether the peripheral clock in certain peripheral tissues of the digestive system could be reset by the feeding clock under RF conditions using *Per1::luc* Tg mice. In this study, the circadian rhythms were compared between the different points of the gastrointestinal tract, the esophagus and the jejunum. The esophagus was reset more strongly under the control of the SCN after 7 days, while the jejunum was entrained not by the SCN, but by the RF. The jejunum was reset for 2–7 days following the RF, unlike the other parts of the gastrointestinal tract. The digestive system tissues were reset by different degrees following RF and were concurrently affected by the central clock.

## 2. Materials and Methods

### 2.1. Animals

The *Per1::luc* Tg mice (kindly provided by Prof. Hajime Tei from the Kanazawa University) were established from a C57BL/6J background, where the *Per1* promoter region (6.8 kb) [7,10] was fused with a luciferase reporter in order to monitor *Per1* expression. *Per1* is a key molecular regulator of the circadian clock; therefore, the molecular rhythms in each tissue of the Tg mice could be estimated as bioluminescent rhythms within a 24 h period. Male mice (*n* = 9) that were twelve weeks to twenty-four weeks old were maintained at 22 °C ± 2 °C, housed in cages with standard pellets (CLEA Rodent Diet CE-2, CLEA, Inc., Tokyo, Japan), and with water available ad lib, except during the RF period. All mice were raised under a 12 h/12 h light–dark cycle (light on 8:00–22:00) at 22 °C in specific-pathogen-free conditions (PAP01B, ORION, Nagano, Japan) for more than 2 weeks until the start of the experiment. All of the animal studies were conducted in accordance with the guidelines of the Committee on Animal Care and Use of the Toyohashi University of Technology (DO2021-1).

#### 2.1.1. Monitoring the Wheel Running Activity Rhythms of *Per1::luc* Tg Mice

**Figure 1:**
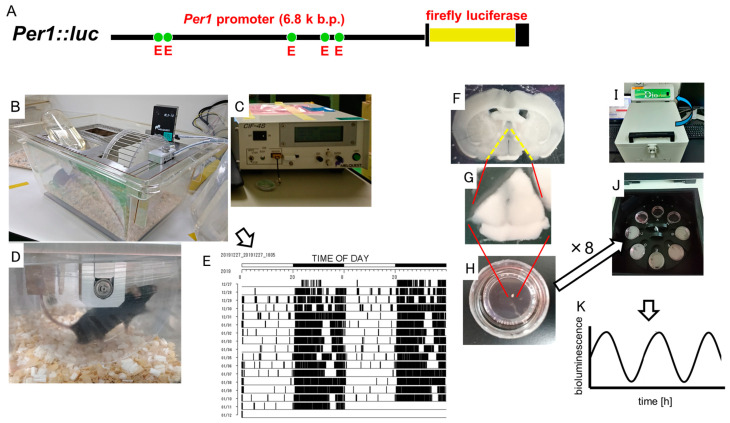
Wheel running unit and luminescence monitoring using Kronos of the cultured SCN tissue in *Per1::luc* Tg mice. (**A**) *Per1::luc* gene: A firefly luciferase gene was fused with the downstream (6.8 kb) promoter region of *Per1*. The yellow box shows the firefly luciferase gene, and the five green circles indicate the E box consensus fragments used to bind with transcriptional factors in the *Per1* promoter region. (**B**) Wheel running unit. WLS-10: wheel running transmitter; RWC-15: wheel rotations. (**C**) Receiver unit. CIF-4S: receiver to count the number of times the wheel was run. (**D**) *Per1::luc* Tg mouse running in the wheel of the cage. (**E**) Double-plot actogram image from Actmaster 4 M software for wheel running analysis. (**F**) Coronal section of the mouse brain slice. (**G**) SCN tissue sections. (**H**) Cultured SCN tissue in a Millicell membrane. (**I**) Kronos illuminometer. (**J**) Turn table of eight sample dishes. (**K**) Rhythmic bioluminescence-like cosine curve.

The locomotor activity of the *Per1::luc* Tg mice was measured in each environmental breeding shelf using a running wheel device (RWC-15, Melquest Ltd., Toyama, Japan) (Figure 1A–D). The mice were habituated to a cage with a rotating wheel, and we measured the wheel running activity for at least 1 week and confirmed them to be acclimated with a stable daily wheel running number before the experiment commenced. The data from the locomotor activity measured with the rotating wheel were analyzed using Actmaster 4 M (Melquest Ltd., Toyama, Japan) and displayed as a double-plot actogram (Figure 1E).

#### 2.1.2. RF (Restricted Feeding) Conditions

**Figure 2:**
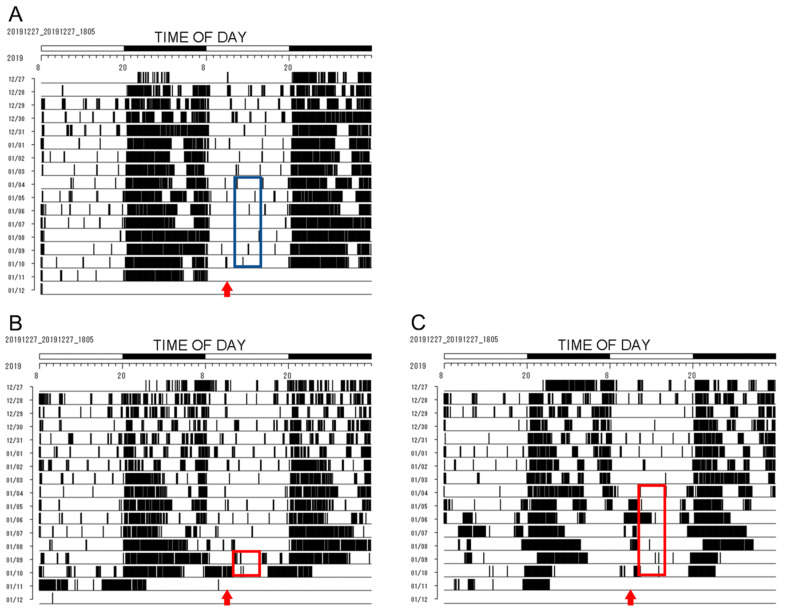
*Per1::luc* Tg mice double-plot actogram of the wheel running rhythms with RF. (**A**) Wheel running rhythms with FF as the control. (**B**) Wheel running rhythms during RF for 2 days. (**C**) Wheel running rhythms during RF for 7 days. Blue frame: the period corresponding to the RF in (**C**). Red frame: the period corresponding to RF; Red arrow: sample preparation time when tissues were removed and cultured from the euthanized *Per1::luc* Tg mice before RF.

The *Per1::luc* Tg mice maintained with FF were subjected to 4 h RF during the rest period under light conditions (Figure 2A). The food was removed from ZT (Zeitgeber Time) 8 on the day before RF, and the mice were fed from ZT 4 to ZT 8 for 4 h from the following day. The RF was continued for 2 or 7 days (*n* = 3) (Figure 2B,C). The mice who received RF for 2 days were euthanized on day 3, and those who received RF for 7 days were euthanized on day 8 at ZT 4.

#### 2.1.3. Monitoring the Circadian Rhythms of Each Tissue of *Per1::luc* Tg Mice

The entire brain, esophagus, and jejunum of the euthanized *Per1::luc* Tg mice were immediately sampled and placed in cooled phosphate-buffered saline (PBS). The cultured tissues were prepared as previously reported [7,10]. The brain was sliced into coronal sections (300 μm thickness) centered on the SCN using a vibratome slicer (Dosaka E.M., Kyoto, Japan), and the SCN and chiasma were sliced into 1.5 mm tissue sections using a scalpel. The peripheral organs were sliced into 1.5 mm square tissue sections using a scalpel (Figure 1E,G). The SCN tissue slices were cultured on Millicell membranes (PICM03050 Merck Millipore, Burlington, MA, USA) (serum-free Dulbecco’s Modified Eagle’s Medium; Thermo Fisher scientific, Waltham, MA, USA) supplemented with 2% B27 (Life Technologies, Carlsbad, CA, USA), 10 mM HEPES (pH 7.2; Invitrogen), 25 unit/mL penicillin, and 25 µg/mL streptomycin (Invitrogen) in a 35 mm dish. The peripheral organ tissues were cultured in a 35 mm dish containing 1.0 mL of the same media, supplemented with 0.1 mM luciferin (Promega) (Figure 1H). The bioluminescent rhythms in the cultured tissues were continuously measured using a Kronos illuminometer (Phot multiple tubes, ATTO, Tokyo, Japan) installed in an incubator and maintained at 36 °C. The emission counts of each cultured tissue were calculated at 1 min intervals over 5 days and plotted on a graph (Figure 1I–K).

The *Per1::luc* emission rhythms were extracted 24–96 h following the tissue preparation and evaluated via cosine fitting using the least-squares method, using NINJA software (ver. 6.0.8.6., Churitsu Electric Corp., Aich, Japan) to calculate the phase and period.

#### 2.1.4. Statistical Analyses

The Tukey–Kramer method in the software R (v.4.1.0., R Foundation for statistical computing, Vienna, Austria) was used to confirm whether there were any statistically significant differences. The statistical significance was set at *p*-values of less than or equal to 0.05, and all analyses were performed with *n* = 3. The error bars on the graph show the mean ± SD.

The *Per1::luc* expression rhythms were analyzed via cosine fitting using NINJA software (ver. 6.0.8.6., CHURITSU Electric, Aich, Japan) and were estimated to display significant circadian oscillation of rhythmic emissions with less than 0.05 error by the least-squares method.

## 3. Results

### 3.1. Wheel Running Activity Rhythms during RF

**Figure 3:**
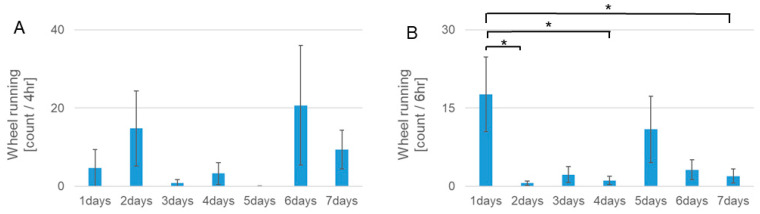
Integrated counts of wheel running of *Per1::luc* Tg mice from 1 day before RF to RF for 7 days. (**A**) Integrated counts of wheel running from 4 h before RF, showing the FAA on each day following RF. FAA increased from day 2 following RF, decreased on days 3–5, and recovered to a high level from day 6 following RF. (**B**) Integrated counts of wheel running in the second half of the active period (6 h). The amount of activity in the latter half of the active period decreased from day 2, increased on days 3–5, and decreased from day 6 onward following RF, in contrast to FAA. *n* = 3. * *p* < 0.05, using the Tukey–Kramer test.

At the onset of RF during the rest period under light conditions, the *Per1::luc* Tg mice in the RF group showed higher activity 2 h before feeding compared with the mice in the FF group (Figure 2). Although the mice in the RF group exercised less on day 1, they exercised on the running wheel device 4 h before feeding on day 2 (Figure 2B). In the 7-day RF group, the amount of exercise decreased 4 h before RF, and in the second half of the active period, because habituation occurred around days 4 and 5 (Figure 2C). On days 6 and 7, a trend toward an increase in the activity rate before the RF was observed. Conversely, in the latter half of the active period during the RF, the amount of exercise of the mice significantly decreased from day 1, except on day 5 (Figure 3A,B).

### 3.2. Monitoring Circadian Rhythms in Each Tissue Sample of the Per1::luc Tg Mice

**Figure 4:**
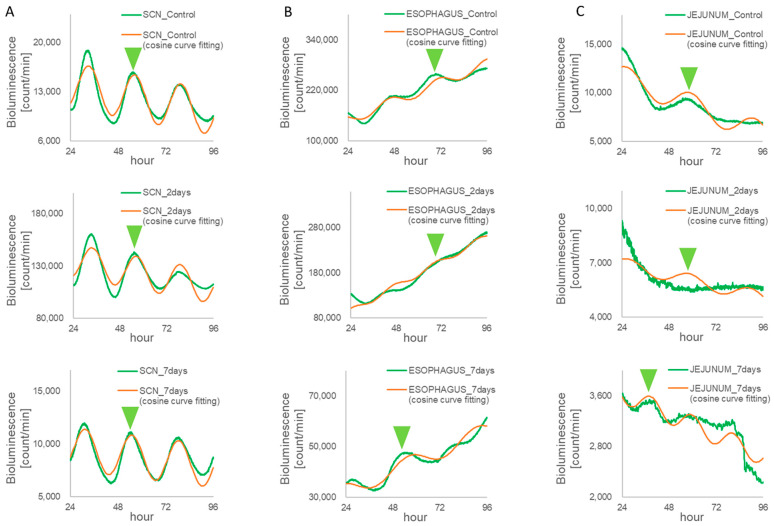
Phase shift of the *Per1::luc* expression rhythm of the SCN and peripheral tissues of the *Per1::luc* Tg mice. The *Per1::luc* expression rhythms from (**A**) the SCN, (**B**) the esophagus, and (**C**) the jejunum for 24–96 h following sample preparation were analyzed via cosine fitting using NINJA software. Amplitudes green triangle: marked on the peak of the cosine curve. The bioluminescence of each point was calculated as the adjacent average of the five points.

We sampled tissue from the *Per1::luc* mice 1 h before feeding time, assuming maximum appetite. The bioluminescence emission rhythms from the tissue sections were monitored in the SCN and the esophagus; however, the amplitude of the emission rhythms in the jejunum tissues was unstable because of a low ability to synchronize the phase of bioluminescence rhythms in all of the cells of the jejunum tissue (Figure 4). In Figure 4, the *Per1::luc* expression rhythms were analyzed via cosine fitting using NINJA software (CHURITSU Electric). The cultured tissue was estimated to display significant circadian oscillation of rhythmic emission if the error levels from a cosine curve by the least-squares method were less than 0.05 on day 7 of the RF. The *Per1* expression rhythms of the SCN, the esophagus, and the jejunum tissues were estimated by using the circadian oscillation of the *Per1::luc* rhythmic emissions, and these phases of the *Per1::luc* expression rhythms were calculated via NINJA. The amplitude rhythm of both peripheral tissues, the jejunum, and the esophagus was lower on day 2 than on day 7 following RF, compared to the SCN, with a high amplitude constitutively. The amplitude of the *Per1::luc* expression rhythms in the jejunum was more unstable with the RF effect than that in the esophagus on day 2 and 7 following RF.

### 3.3. Phase Shift of the Bioluminescent Emission Rhythms during RF

**Figure 5:**
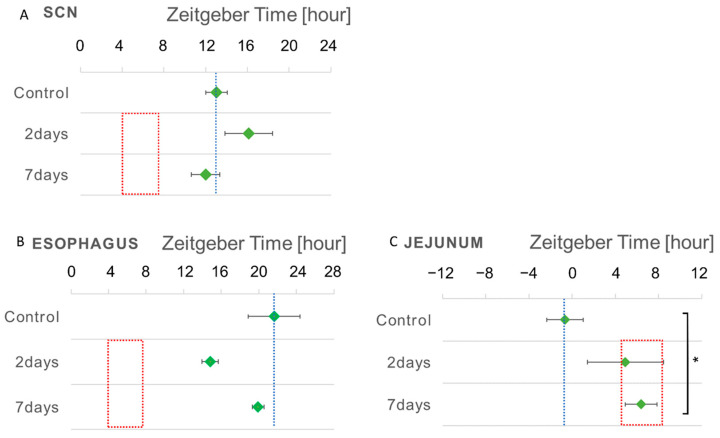
Phase shift of the *Per1::luc* expression rhythms of the SCN and peripheral tissues during RF for 2 and 7 days. Green points: the peak time of the *Per1::luc* expression rhythms of (**A**) the SCN, (**B**) the esophagus, and (**C**) the jejunum tissues in the FF mice; control, RF for 2 days, and RF for 7 days. Red frame: RF period. Blue line: the peak position in FF as the control. *n* = 3. * *p* < 0.05, using the Tukey–Kramer test.

Little phase shift was detected in the SCN tissues that were isolated from the *Per1::luc* Tg mice following RF. However, phase shifts in the bioluminescent emission rhythms of the peripheral tissues in the 2- and 7-day RF groups were unchanged compared with those of the FF control group (Figure 5A, Table 1). The peripheral tissues, including the esophagus and the jejunum, demonstrated shifted *Per1* gene expression peaks in response to the feeding time (Figure 5B,C, Table 1). The *Per1* peak time of the esophagus returned to that of the control FF (Figure 5B). The esophagus exhibited a phase delay of ~7 h following the RF for 2 days, whereas the RF for 7 days produced a phase advance of ~2 h, compared with that of the control FF. On the other hand, the *Per1* peak time in the jejunum maintained the shift of the phase delay by 7 h following the RF for both 2 and 7 days (Figure 5C).

An RF-induced phase shift was hardly observed in the SCN tissues following the RF for 2 and 7 days; however, in the peripheral tissues, a clear phase shift from 2 to 7 h could be observed following the RF for 2 days.

In the 7-day RF group, the peak time of the esophagus tended to return to the peak time of the FF control of ~5 h; however, this return was not observed in the jejunum. This was because the photic clock under the light–dark conditions was more strongly entrained in the 7-day RF group than in the 2-day RF group. Therefore, it is reasonable to assume that the amount of wheel running activity increased 2 h before the RF in the 2-day RF group, but decreased in the 7-day RF group under LD conditions, with the control of the photic clock in the SCN.

## 4. Discussion

After the *Per1::luc* Tg mice were maintained under FF 12 h/12 h light–dark cycle conditions, they underwent RF for 4 h during the rest period in the light conditions. Subsequently, we observed the phase shifts of the bioluminescent rhythms of the different points of the gastrointestinal tract, the esophagus and the jejunum, and the cultured SCN, of the *Per1::luc* Tg mice.

The *Per1::luc* Tg mouse biosensors have several advantages in terms of the ability to reset the rhythm in each peripheral tissue in response to changing environmental signals. As previously described, the *Per1::luc* Tg mice allowed us to observe the circadian rhythms over a specific time course using consecutive data from a bioluminescent rhythmic curve without individual variation.

The biosensors of the *Per1::luc* Tg mice enable the molecular clock of *Per1* gene expression to be monitored in real time as a continuous luminescence rhythm over a 24 h period, which can be analyzed by fitting the data to a cosine curve. Another advantage of simulating the cosine curve data is the ease with which the rhythmic changes can be estimated and predicted. Furthermore, a luciferase reporter is suitable for long-term stable observation. In order to observe a phase of the circadian rhythm, data from at least three consecutive days are required; moreover, the phototoxicity of the excitation light must be considered when performing a quantitative analysis using a fluorescent reporter.

The bioluminescent rhythms of the SCN tissues remained unchanged in the RF groups compared to the FF control group, whereas the bioluminescent rhythms of the peripheral tissues demonstrated a phase shift of 4–9 h in the 2- and 7-day RF groups compared to the FF control group. In a previous report, the stomach and the colon of the digestive system could be reset with a 4 h RF period for 2 days and returned to a nocturnal peak when the mice were kept with ad lib (FF) for ~5 days, because the stomach and the colon can easily return to under the control of the SCN [19]. In this study, the circadian rhythms were compared between the different points of the gastrointestinal tract, the esophagus and the jejunum. The esophagus was reset more strongly under the control of the SCN after 7 days, while the jejunum was entrained not by the SCN, but by the RF. The jejunum was reset for 2–7 days in the RF, unlike the other parts of the gastrointestinal tract. The jejunum secretes some of the intestinal fluids, but it is unclear how the different entrained abilities of the gastrointestinal tract could contribute to the digestion of food. In this study, the peak time of the esophageal tissues tended to return to that of the FF control group, unlike that of the jejunum tissues in the 7-day RF group. Therefore, the esophagus was more strongly regulated under the control of the SCN than the jejunum. The effect of the central clock on the peripheral tissues was also unclear, including whether it has any relationship with the different functions in digestion and/or the stability of the peripheral clock; however, it seems likely that the feeding clocks can regulate each peripheral clock of the digestive system independently of the SCN following a few days of RF.

The peak phases of the emission rhythms were in the peripheral tissue, except for the SCN, which did not shift toward the period of the RF (Figure 5, red line period). The different peripheral tissues of the digestive system were entrained in different time zones of the RF, such as the jejunum after RF, in accordance with their period of function after food digestion, based on the feeding clock. In contrast, the esophagus may not be strongly entrained because it may not be directly involved in digestion or may function over time during a meal. Therefore, the feeding clock entrains the peripheral tissues of the digestive system to varying extents.

**Figure 6:**
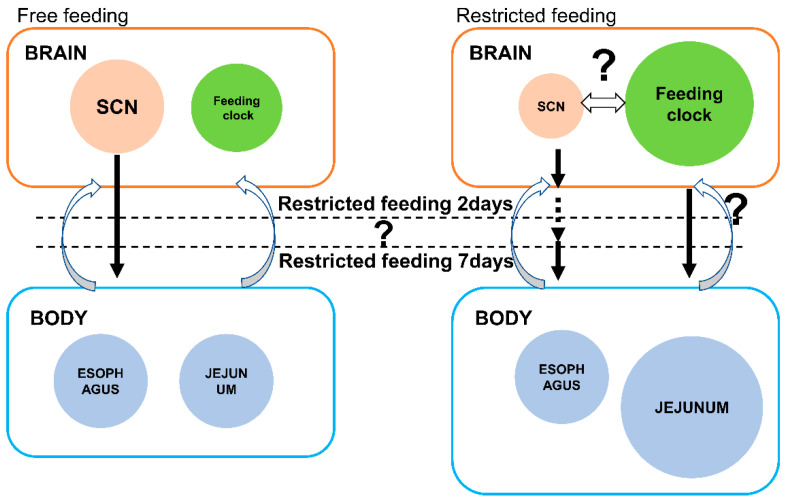
Schematic presenting the light and feeding clocks. The photic clock of the SCN and the feeding clock are located above the peripheral tissue in the hierarchy of the circadian rhythms. The black arrows indicate the upper clock in the brain, which regulates the rhythms in the peripheral clock on the body. The broken arrows show weaken effect in 4 h RF for 2 days The straight white arrow indicates the coupling between the SCN and feeding clock during RF. The curved white arrows from the bottom to the top represent the peripheral clock feedback to the upper clock.

Figure 6 presents a schematic model of the association between the photic clock (located in the SCN) and the feeding clock (at an unknown location) in the hierarchy of the circadian rhythms in the FF and RF groups. In the FF control, the photic clock was reset by a photic signal and entrained the other clocks of the peripheral tissues, whereas, in the RF group, the feeding clock was reset by the fasting signal, and this entrained each clock in the peripheral tissues of the digestive system. Although the effect of the feeding clock was greater than that of the photic clock in the RF group for 2 days in the esophagus, the effect of the photic clock was greater than that of the feeding clock iin the RF group for 7 days in a reversed and gradual manner.

It is reasonable to presume that the peripheral tissues, whose bioluminescent rhythm phase is reset by RF, correspond to the tissues in the digestive system. The nonphotic signal and feeding time reset the digestive system rhythms, which are directly related to the phase of the circadian rhythms in the SCN and are strongly synchronized with the light–dark cycle of the external environment, but not with the nonphotic RF signals.

In order to adjust the circadian rhythm within the whole body, it is advantageous for the organism to have two types of clocks: light and feeding clocks (Figure 6). By providing several windows to various external environmental signals, an organism can respond more flexibly to changes in the environment. In terms of organisms that survive in static environments, such as in polar or ocean abyssal regions where the sunlight does not reach, they may adjust their feeding clock to consume food at a constant rate.

There are also the questions of whether the SCN is affected by the feedback signals of the feeding clock, and which mechanisms of the feeding clock are independent of the SCN. In order to elucidate the answers to these questions in the future, external experiments are necessary.

## 5. Conclusions

Our physiological activity conforms to the circadian rhythm over a 24 h period that is tuned to Earth’s rotation period. In the SCN of mammals, the photic central oscillator predominates over all of the peripheral tissues at the top of the circadian rhythm hierarchy. Conversely, the nonphotic oscillator based on the fasting signals from the feeding time becomes independent of the SCN. Previously, we established *Per1::luc* Tg mice to monitor, in real time, the phase of the circadian rhythms in each tissue using bioluminescent rhythms. Using this system, we examined whether 4 h RF during the rest period under light conditions could entrain the central clock and the peripheral clock of the digestive system. Bioluminescent rhythms were observed in the SCN, the esophagus, and the jejunum tissues of Tg mice in RF groups for 2 and 7 days. The peak time of *Per1::luc* emission in the SCN was not affected by the feeding time condition and was maintained under the FF state as the standard oscillator of the light–dark cycle. While both peak times in the esophagus and the jejunum exhibited a phase shift of ~7 h following RF for 2 days, the peak time in the esophagus returned to that of the FF control at 7 days, unlike that in the jejunum. According to comparisons between the esophagus and the jejunum in the gastrointestinal tract as one long organization, each peripheral clock of the digestive system can entrain the rhythms through the fasting signal that is induced by RF, independent of the SCN. It is possible that this varying ability to entrain to the feeding time in the digestive system tissues provides an advantage for food digestion and metabolism.

## Figures and Tables

**Table 1:** Phase shift in restricted feeding (RF).

Tissue	RF for 2 Days	RF for 7 Days
SCN	3.1 h (Phase Delay)	−1.0 h (Phase Advance)
Esophagus	−6.8 h (Phase Advance)	−1.7 h (Phase Advance)
Jejunum	5.6 h (Phase Delay)	7.0 h (Phase Delay)

## Data Availability

The data presented in this study are available in supplementary material.

## Supplementary Materials

The following supporting information can be downloaded at: https://www.mdpi.com/article/10.3390/biomedicines11051463/s1.

## References

[1] Tei H., Okamura H., Shigeyoshi Y., Fukuhara C., Ozawa R., Hirose M., Sakaki Y. (1997). Circadian oscillation of a mammalian homologue of the Drosophila period gene. Nature.

[2] Sun Z.S., Albrecht U., Zhuchenko O., Bailey J., Eichele G., Lee C.C. (1997). RIGUI, a Putative Mammalian Ortholog of the Drosophila period Gene. Cell.

[3] Shearman L.P., Zylka M.J., Weaver D.R., Kolakowski L.F., Reppert S.M. (1997). Two period Homologs: Circadian Expression and Photic Regulation in the Suprachiasmatic Nuclei. Neuron.

[4] Siepka S.M., Yoo S.-H., Park J., Lee C., Takahashi J.S. (2007). Genetics and Neurobiology of Circadian Clocks in Mammals. Cold Spring Harb. Symp. Quant. Biol..

[5] Shigeyoshi Y., Taguchi K., Yamamoto S., Takekida S., Yan L., Tei H., Moriya T., Shibata S., Loros J.J., Dunlap J.C. (1997). Light-induced resetting of a mammalian circadian clock is associated with rapid induction of the mPer1 transcript. Cell.

[6] Numano R., Yamazaki S., Umeda N., Samura T., Sujino M., Takahashi R.-I., Ueda M., Mori A., Yamada K., Sakaki Y. (2006). Constitutive expression of the *Period1* gene impairs behavioral and molecular circadian rhythms. Proc. Natl. Acad. Sci. USA.

[7] Yamazaki S., Numano R., Abe M., Hida A., Takahashi R.-I., Ueda M., Block G.D., Sakaki Y., Menaker M., Tei H. (2000). Resetting Central and Peripheral Circadian Oscillators in Transgenic Rats. Science.

[8] Page A.J. (2021). Gastrointestinal Vagal Afferents and Food Intake: Relevance of Circadian Rhythms. Nutrients.

[9] Grosjean E., Simonneaux V., Challet E. (2023). Reciprocal Interactions between Circadian Clocks, Food Intake, and Energy Metabolism. Biology.

[10] Stokkan K.-A., Yamazaki S., Tei H., Sakaki Y., Menaker M. (2001). Entrainment of the Circadian Clock in the Liver by Feeding. Science.

[11] Stephan F. (1984). Phase shifts of circadian rhythms in activity entrained to food access. Physiol. Behav..

[12] Stephan F.K. (1992). Resetting of a feeding-entrainable circadian clock in the rat. Physiol. Behav..

[13] Davidson A.J., Aragona B.J., Werner R.M., Schroeder E., Smith J.C., Stephan F.K. (2001). Food-anticipatory activity persists after olfactory bulb ablation in the rat. Physiol. Behav..

[14] Damiola F., Minh N.L., Preitner N., Kornmann B., Fleury-Olela F., Schibler U. (2000). Restricted feeding uncouples circadian oscillators in peripheral tissues from the central pacemaker in the suprachiasmatic nucleus. Genes Dev..

[15] Balsalobre A., Brown S.A., Marcacci L., Tronche F., Kellendonk C., Reichardt H.M., Schütz G., Schibler U. (2000). Resetting of Circadian Time in Peripheral Tissues by Glucocorticoid Signaling. Science.

[16] Davidson A.J., Castanon-Cervantes O., Leise T.L., Molyneux P.C., Harrington M.E. (2009). Visualizing jet lag in the mouse suprachiasmatic nucleus and peripheral circadian timing system. Eur. J. Neurosci..

[17] Yamaguchi S., Isejima H., Matsuo T., Okura R., Yagita K., Kobayashi M., Okamura H. (2003). Synchronization of Cellular Clocks in the Suprachiasmatic Nucleus. Science.

[18] Nagano M., Adachi A., Nakahama K.-I., Nakamura T., Tamada M., Meyer-Bernstein E., Sehgal A., Shigeyoshi Y. (2003). An Abrupt Shift in the Day/Night Cycle Causes Desynchrony in the Mammalian Circadian Center. J. Neurosci..

[19] Davidson A.J., Poole A.S., Yamazaki S., Menaker M. (2003). Is the food-entrainable circadian oscillator in the digestive system?. Genes Brain Behav..

[20] Pendergast J.S., Oda G.A., Niswender K.D., Yamazaki S. (2012). *Period* determination in the food-entrainable and methamphetamine-sensitive circadian oscillator(s). Proc. Natl. Acad. Sci. USA.

[21] Pendergast J.S., Yamazaki S. (2018). The Mysterious Food-Entrainable Oscillator: Insights from Mutant and Engineered Mouse Models. J. Biol. Rhythm..

[22] Pendergast J.S., Yamazaki S. (2014). Effects of light, food, and methamphetamine on the circadian activity rhythm in mice. Physiol. Behav..

[23] Pendergast J.S., Wendroth R.H., Stenner R.C., Keil C.D., Yamazaki S. (2017). mPeriod2 Brdm1 and other single Period mutant mice have normal food anticipatory activity. Sci. Rep..

[24] Sugiyama M., Nishijima I., Nakamura W., Nakamura T.J. (2022). Secretin receptor-deficient mice exhibit robust food anticipatory activity. Neurosci. Lett..

[25] Pendergast J.S., Nakamura W., Friday R.C., Hatanaka F., Takumi T., Yamazaki S. (2009). Robust Food Anticipatory Activity in BMAL1-Deficient Mice. PLoS ONE.

[26] Takasu N.N., Kurosawa G., Tokuda I.T., Mochizuki A., Todo T., Nakamura W. (2012). Circadian Regulation of Food-Anticipatory Activity in Molecular Clock–Deficient Mice. PLoS ONE.

